# Freeze-Dried Matrices Composed of Degradable Polymers with Surfactant-Loaded Microparticles Based on Pectin and Sodium Alginate

**DOI:** 10.3390/ma14113044

**Published:** 2021-06-03

**Authors:** Natalia Stachowiak, Jolanta Kowalonek, Justyna Kozlowska

**Affiliations:** Faculty of Chemistry, Nicolaus Copernicus University in Torun, Gagarina 7, 87-100 Torun, Poland; jolak@umk.pl (J.K.); justynak@umk.pl (J.K.)

**Keywords:** sodium alginate, pectin, microparticles, surfactant, porous matrix, degradable polymer

## Abstract

Gelatin/polyvinylpyrrolidone/hydroxyethyl cellulose/glycerol porous matrices with microspheres made of sodium alginate or pectin and sodium alginate were produced. A surfactant was loaded into these microparticles. The microspheres were characterized using optical microscopy, scanning electron microscopy SEM, and laser diffraction particle size analyzer. For the matrices, the density, porosity, swelling capacity, dissolution in phosphate saline buffer were determined and SEM, mechanical, and thermogravimetric studies were applied. The results showed that the size of the two-component microspheres was slightly larger than that of single-ingredient microparticles. The images confirmed the spherical shape of the microparticles. The prepared matrices had high water uptake ability and porosity due to the presence of hydrophilic polymers. The presence of microparticles in the matrices caused a decrease in these parameters. Degradation of the composites with the microspheres was significantly faster than the matrix without them. The addition of microparticles increased the stiffness and toughness of the prepared materials. The efficiency of the thermal decomposition main stage was reduced in the samples with microspheres, whereas a char residue increased in these composites.

## 1. Introduction

The sponge-like matrices have many advantages, including high porosity, specific surface area, and primarily, a simple preparation procedure, which is a freeze-drying method involving solvent sublimation [1,2]. As a result, a porous, three-dimensional solid structure with appropriate flexibility is fabricated [3,4]. Several papers have reported on the preparation and characteristics of sponges produced from biodegradable natural or synthetic polymers for various applications. Sponge-like dressings based on chitosan and glycosaminoglycans (GAGs) filled with tranexamic acid were produced. Such materials combine hemostatic and proliferative properties and showed promise in bleeding control and wound healing as well as for abdominal surgery [5]. The gelatin scaffolds were fabricated and crosslinked with glutaraldehyde (GTA), genipin (GP), 1-ethyl-3-(3-dimethyl aminopropyl)carbodiimide (EDC), and microbial transglutaminase (mTG). The mTG–matrix is a suitable biomaterial for repairing most soft tissues as it is characterized by good water absorption, flexibility, and biocompatibility. On the other hand, the genipin cross-linked sponge can be used for hard tissue repair because it exhibits lower water absorption, larger pore size, and higher mechanical strength [6]. The three-dimensional collagen scaffold provided support for cancer cell growth, which is a physiologically appropriate biomedical research tool and preclinical drug testing [7]. Moreover, the appropriate combination of hyaluronic acid (HA), silk fibroin (SF), and heparin form aerogel scaffolds with synergistic features in terms of cell infiltration (HA), mechanical properties (SF), and heparin release control [8]. Furthermore, nanocomposite sponges based on sodium alginate/graphene oxide (GO)/polyvinyl alcohol loaded with norfloxacin (NFX) were produced. These materials exhibited suitable water absorption and breathability to maintain the moisture balance of the wound and sustained release of NFX. In vitro cytocompatibility studies demonstrated that the nanocomposites were non-toxic toward NIH 3T3 cells, and the presence of GO could promote cell proliferation [9]. The carboxymethyl cellulose sponges can be applied to remove heavy metal ions due to their unique properties such as reusability, high adsorption capacity, and good mechanical and thermal stability [10].

Drugs or other biologically active substances can be encapsulated in microparticles. The microparticles tend to be spherical particles ranging in size from 1 to 1000 µm [11]. They are composed of natural polymers (sodium alginate [12], carrageenan [13], chitosan [14], and gellan gum [15]), and synthetic ones (polylactide [16], and poly (lactic-co-glycolic acid) (PLGA) [17], and polycaprolactone [18]. The microparticles can be divided into two groups: microspheres and microcapsules [19]. The essential difference between the microspheres and the microcapsules is based on the method of active agent introduction. In microspheres, it is loaded by dissolution or suspension in a polymer matrix, whereas in microcapsules, it is entrapped in a liquid or solid form within the polymeric shell [20]. The microparticles are extensively applied in medical and pharmaceutical industries as effective carriers of encapsulated drugs in the drug delivery system [21,22]. For this purpose, particles with a narrow size distribution are desirable [23]. The attention of researchers to microparticles has increased due to their specific features, including high surface-to-volume ratio, relatively low-cost production, and especially the possibility to load various substances and control the rate of release [24,25]. The microparticles consisting of internal chitosan core and a surface layer of poly-(styrene-co-maleic anhydride) (PSMA) loaded with bovine serum albumin as a model drug demonstrated a reduction of initial burst and cumulative release. Moreover, the obtained carriers showed limited solubility under acidic conditions and enzymatic degradation, which allows them to be used in the acidic environment, such as the human stomach [26]. Moreover, the konjac glucomannan microparticles containing two drugs, isoniazid (INH) and rifabutin (RFB), were developed to antitubercular therapy for the pulmonary delivery system [27]. The PLGA microparticles incorporated with rosmarinic acid had antioxidant and antibacterial activity. They can be applied in cosmetic and pharmaceutical products due to their non-toxicity for human dermal fibroblasts [28]. Several types of processes are commonly used for the fabrication of polymer microparticles, including complex coacervation [29], single/double emulsion method [30,31], spray drying [32], solvent extraction/evaporation [33], and extrusion [34].

In this experiment, an encapsulation machine was used for microspheres creation. This easy-to-use technology relies on the laminar jet break up of a liquid stream into uniformly sized droplets by applying a controlled vibration frequency to the liquid [35]. The method involves mixing a polymer solution with an active substance. The mixture extruded through a nozzle of appropriate diameter is dropped into the vessel with the crosslinking agent solution, in which microspheres are formed [36]. The microspheres’ size is approximately twice the diameter of the used nozzle. The manufacture of microparticles is controlled by several parameters, i.e., vibration frequency, liquid flow rate, and electrode tension. It enables various individual settings and adjusts the size and shape of the produced microspheres [37].

It should be mentioned that the properties of the microparticles, e.g., size, shape, loading efficacy, surface properties, and colloidal stability, can be improved by adding a surfactant during the preparation process [38]. Surfactants, in other words surface-active agents, consist of nonpolar long carbon chains with a polar head. The hydrophobic part interacts with fat-soluble ingredients, while the hydrophilic part attracts water-soluble components [39,40]. Surfactants have the capability to reduce the intermolecular forces, thus lowering the surface or interfacial tension [41]. Surfactants are categorized according to their charge, i.e., anionic, cationic, non-ionic, and amphoteric (negative and positive charge) [42]. Alkyl glucosides, such as coco-glucoside and decyl glucoside, are non-ionic surfactants synthesized by the condensation of long-chain fatty alcohols and glucose [43]. Owing to the fact that the alkyl glucosides are extracted from natural, renewable sources, they are characterized by biodegradability and low toxicity [44]. Alkyl glucosides display washing, foaming, and emulsifying properties [45].

To the best of our knowledge, there is no report on the usage of polymeric sponge-like matrices as a substitute for wet wipes. Moreover, the incorporation of surfactant-loaded microparticles into such materials intended for cleansing products has not been studied yet. The aim of this research was to obtain and characterize materials with potential use as wet wipes due to the presence of washing agents. For this purpose, microparticles with the entrapped surfactant (coco-glucoside) were placed in the 3D polymeric matrix. The surfactant release could occur as a result of the degradation of the particles upon immersion in water. Polysaccharides (sodium alginate, pectin, and hydroxyethyl cellulose), protein (gelatin), and synthetic water-soluble polymer (polyvinylpyrrolidone) used for the preparation of the matrices and particles were non-toxic and degradable.

## 2. Materials and Methods

### 2.1. Materials

Gelatin type A from porcine skin (GEL), polyvinylpyrrolidone (PVP), hydroxyethyl cellulose (HEC), pectin from the citrus peel (P), 1-ethyl-3(3-dimethylamino propyl) carbodiimide (EDC), and N-hydroxysuccinimide (NHS) were purchased from Sigma-Aldrich (Poznan, Poland). Sodium alginate (ALG) was supplied by BÜCHI Labortechnik AG (Flawil, Switzerland). Glycerol (G) was supplied by Stanlab (Lublin, Poland). Coco-Glucoside (CG) was acquired from Greenaction (Kielce, Poland). All used chemicals were analytical grade.

### 2.2. Microparticles Preparation

**Figure 1:**
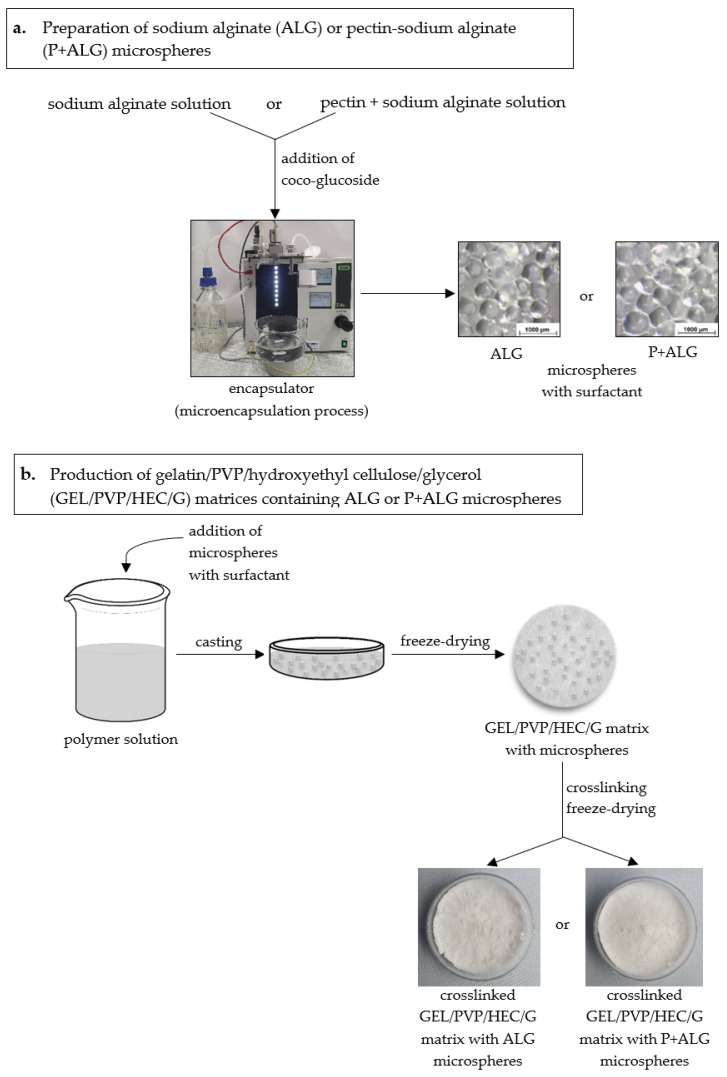
The scheme of preparation of sodium alginate and pectin-sodium alginate microspheres (**a**) and production of gelatin/PVP/hydroxyethyl cellulose/glycerol matrices with microspheres (**b**).

Microparticles (M) consisting of sodium alginate or a mixture of pectin and sodium alginate were prepared using an encapsulator (B-395 Pro, BÜCHI Labortechnik AG, Flawil, Switzerland) (Figure 1a) [46]. In order to obtain sodium alginate microparticles M(ALG), a 1.5% (*w*/*w*) aqueous solution of sodium alginate containing 1% of a non-ionic surfactant (Coco-Glucoside) was prepared and transferred into a pressure bottle. Then, the mixture was forced through a 300 µm diameter nozzle and separated into droplets by an electrical field. The microparticles formation process occurred in the bath with the crosslinking agent solution (0.5 M CaCl_2_), which was continuously mixed by a magnetic stirrer to prevent microparticles clumping. The collected microparticles were rinsed with distilled water and dried at room temperature for 3 h. An analogous procedure was used to obtain pectin-sodium alginate microparticles M(P + ALG). For this purpose, the mixture of 2% pectin and 0.5% sodium alginate solution containing 1% of the surfactant was made.

### 2.3. Characterization of Microparticles

#### 2.3.1. Microparticles Imaging

The appearance of the prepared microparticles was observed by the optical microscope Motic SMZ-171 BLED (Hong Kong, China). Imaging of swollen, dry, and rehydrated microspheres were performed. Drying and rehydration of the samples took 24 h.

#### 2.3.2. Particle Size Distribution Analysis

Particle size and size distribution of the obtained microparticles were measured by a laser diffraction particle size analyzer (SALD-2300, Shimadzu, Kyoto, Japan) equipped with a sampler (SALD-MS23, Shimadzu, Kyoto, Japan). This device is capable of measuring particle size in the range of 17 nm to 2500 µm. The particle size distribution was determined by the light intensity distribution pattern of scattered light generated by a sample irradiated with a laser. The specimen was mixed with distilled water in the dissipation bath. Next, it was circulated through the flow cell in the measuring unit and irradiated with the laser beam [47]. The results were recorded by Wing SALD II software (version 3.1.0, Shimadzu, Kyoto, Japan). The size distribution was evaluated with the span value calculated using the equation:

Span = (X_90_ − X_10_)/X_50_ (1)

where X_10_, X_50_, and X_90_ represent the volume percentages of the particles (10%, 50%, and 90% undersize, respectively). The span value is an index of polydispersity of microparticles [48].

### 2.4. Matrices Preparation

The porous polymer matrices were produced by the freeze-drying technique. The scheme of these materials production was shown in Figure 1b. First, a polymer solution consisting of gelatin (2.0 g), PVP (0.8 g), hydroxyethyl cellulose (0.8 g), glycerol (1.4 g), and water (95 mL) was prepared to obtain microparticles-loaded matrices. Then, the microparticles (2.0 g) were added to 20 mL of the mixtures and magnetically stirred for 30 min. The prepared mixtures with the microparticles were poured into Petri dishes (5 cm diameter), frozen (−18 °C, 24 h), and lyophilized (−55 °C, 5 Pa, 24 h). After lyophilization, the matrices were crosslinked with 1-ethyl-3(3-dimethylamino propyl) carbodiimide (EDC) and N-hydroxysuccinimide (NHS), according to a protocol described previously [49]. Gelatin/PVP/hydroxyethyl cellulose/glycerol matrix containing sodium alginate microparticles was named GEL/PVP/HEC/G + M(ALG), and the other material incorporating pectin-sodium alginate microparticles was named GEL/PVP/HEC/G + M(P + ALG). The matrix without microspheres was used as a control sample (GEL/PVP/HEC/G).

### 2.5. Characterization of Matrices

#### 2.5.1. Structure and Morphology of Materials

Scanning electron microscopy (SEM) imaging was conducted using the scanning electron microscope (Quanta 3D FEG, Quorum Technologies, Lewes, UK) to analyze the structure of the porous matrices with and without microparticles. SEM images of microspheres loaded into matrices along with their surface were also taken. Before the analysis, the sample surface was sprayed with a thin layer of gold and palladium.

#### 2.5.2. Determination of Porosity and Density

The porosity (Є) and the density (*d*) of the prepared materials were estimated by the liquid displacement technique as early reported [50]. Isopropanol was used in these measurements as a nonsolvent of matrix-forming polymers. The sample was weighted (W) and immersed in a graduated cylinder containing a specific volume of isopropanol (V_1_). After 5 min, the liquid volume (V_2_) was recorded. The isopropanol-impregnated matrix was removed from the cylinder, and the residual isopropanol volume (V_3_) was noted. Each sample was measured in triplicate. The porosity and the density of the matrices are expressed as follows:

Є (%) = (V_1_ − V_3_)/(V_2_ − V_3_) × 100 (2)

*d* = W/(V_2_ − V_3_) (3)

#### 2.5.3. Evaluation of Swelling Ability

The swelling capacity of the obtained materials was determined after immersing them in a phosphate saline buffer (PBS) at pH 5.7 for 3 h. Three specimens of each dried porous matrix were weighed (W_1_) and submerged in PBS solution. The measurements were carried out after 15 min, 30 min, 1 h, 2 h, and 3 h. After each time, the samples were removed from phosphate saline buffer and weighted (W_2_) [51]. The swelling ratio of matrices was defined according to the following equation:

swelling ratio (%) = (W_2_ − W_1_)/W_1_ × 100 (4)

#### 2.5.4. Dissolution of Matrices

The prepared matrices were weighed (W_b_) and submerged in phosphate saline buffer (pH = 5.7). The specimens were incubated at room temperature 1, 2, 3, 7, 14, 21, and 28 days. After each period, they were taken out of the PBS solution and washed with deionized water three times. Then, the samples were frozen, lyophilized, and reweighed (W_a_) [52]. The percentage weight loss was calculated according to the following equation:

weight loss [%] = (W_b_ − W_a_)/W_b_ × 100 (5)

#### 2.5.5. Mechanical Tests

Mechanical properties of the obtained matrices were conducted at room temperature using a mechanical testing machine equipped with compression jigs (EZ-Test SX Texture Analyzer, Shimadzu, Kyoto, Japan). Before the experiment, the diameters of cylindrical samples were measured. The dry samples and the samples soaked in water for 1 min were examined. The tests were carried out at a compression speed of 5 mm/min up to 60% of strain. The elastic modulus (Young’s modulus, E) was calculated from the slope of the stress–strain curve in the linear region (strain from 0.07% to 0.20%). Toughness, defined as the amount of absorbed energy per unit volume up to 60% of the sample strain, was calculated. Toughness is related to the area under the stress–strain curve [53]. Yield strength of the dry samples was also determined. The yield strength refers to stress at the yield point on the stress–strain curve from which plastic deformations appear in the studied material. The results were recorded by Trapezium X software (version 1.4.5, Shimadzu, Kyoto, Japan). The presented data are the average values calculated from five measurements for each type of matrices.

#### 2.5.6. Thermal Analysis

Thermal stability of the prepared materials was performed by the thermogravimetric instruments (SDT 2960, TA Instruments, New Castle, DE, USA). The measurements were conducted in a nitrogen atmosphere with a heating rate of 10 °C/min up to 600 °C. From thermogravimetric (TG) and derivative thermogravimetric (DTG) curves, characteristic parameters were determined: T_0_ (°C)—the temperature at the beginning of the sample decomposition; T_max_ (°C)—the temperature at the maximum degradation rate (maximum on DTG curve); Δm (%)—weight loss in the main process; and a char residue (%) at 600 °C.

## 3. Results and Discussion

### 3.1. Microparticles Characterization

**Figure 2:**
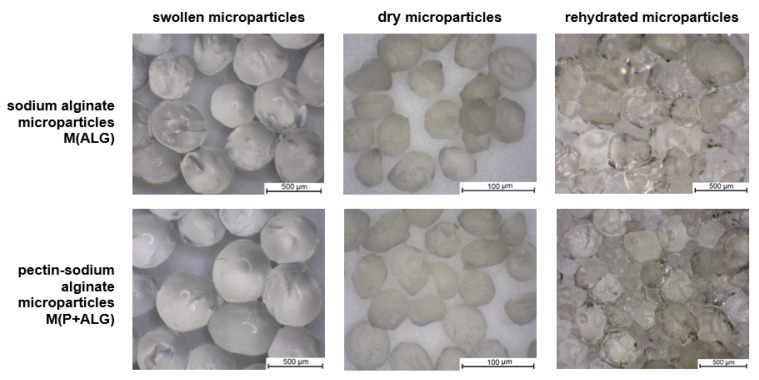
Optical microscope images of sodium alginate and pectin-sodium alginate microparticles containing surfactant for swollen, dry, and rehydrated particle forms.

The appearance of the polymer microparticles containing surfactant is presented in Figure 2. Based on the optical microscope images, no effect of the microsphere composition on their surface and shape were detected. Swollen microspheres had a spherical shape and smooth surface. By contrast, the microspheres changed their shape and size after drying. Dried microparticles exhibited rough surfaces with evident cavities. In addition, rehydrated microparticles swelled, increasing in size but not returning to their previous dimensions. Moreover, they revealed an irregular shape and ridged surface with dimples after water re-immersion. Vreeker et al. studied the rehydration properties of air-dried calcium alginate gel beads. They indicated that the poor rehydration properties of these beads in pure water are attributed to egg-box multimer structures formed during drying [54].

**Figure 3:**
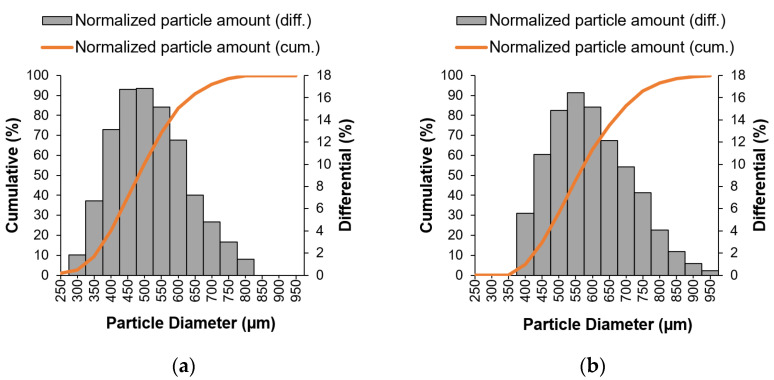
Particle size distribution for sodium alginate (**a**) and pectin-sodium alginate (**b**) microparticles containing surfactant.

Particle size analysis results are shown in Figure 3 and Table 1. The particle size and the size distribution of the microparticles were determined by a laser diffraction method in wet conditions. The microparticles were prepared using the encapsulator with a 300 µm nozzle diameter.

On the basis of the obtained results (Table 1), the sodium alginate and pectin-sodium alginate microparticles presented size distributions in the ranges 300–800 µm and 400–950 µm, respectively. It can be noted that microparticle composition affects the particle size distribution (Figure 3). The data demonstrate that although the surface and shape of the particles displayed similar characteristics, they possessed distinct size distribution. The mean particle size of M(ALG) and M(P + ALG) were about 475 µm and 560 µm, respectively. The microparticles consisting of sodium alginate had a smaller mean particle size and slightly higher span value than the microspheres based on pectin and sodium alginate. Alginates and pectin possess carboxylate groups capable of forming ionic interactions with calcium cations, leading to a crosslinked structure. However, alginates can create a special structure egg-box because the size of calcium cations is the same as the space between the chain fragments, and these ions are placed in the crosslinked network [54,55]. It may explain the smaller sizes of M(ALG) compared to M(P + ALG). Furthermore, in pectin chains, some carboxylic groups can be esterified, which reduces the number of carboxylic groups able to create ionic bonds with cations. Thus, the combination of pectin and sodium alginate allows the formation of larger microparticles and also more homogeneity. Nevertheless, low span value is related to narrow particle size distribution. Such low polydispersity is common for microparticles produced using an encapsulation machine [35].

### 3.2. Materials Characterization

#### 3.2.1. Structure and Morphology of Materials

**Figure 4:**
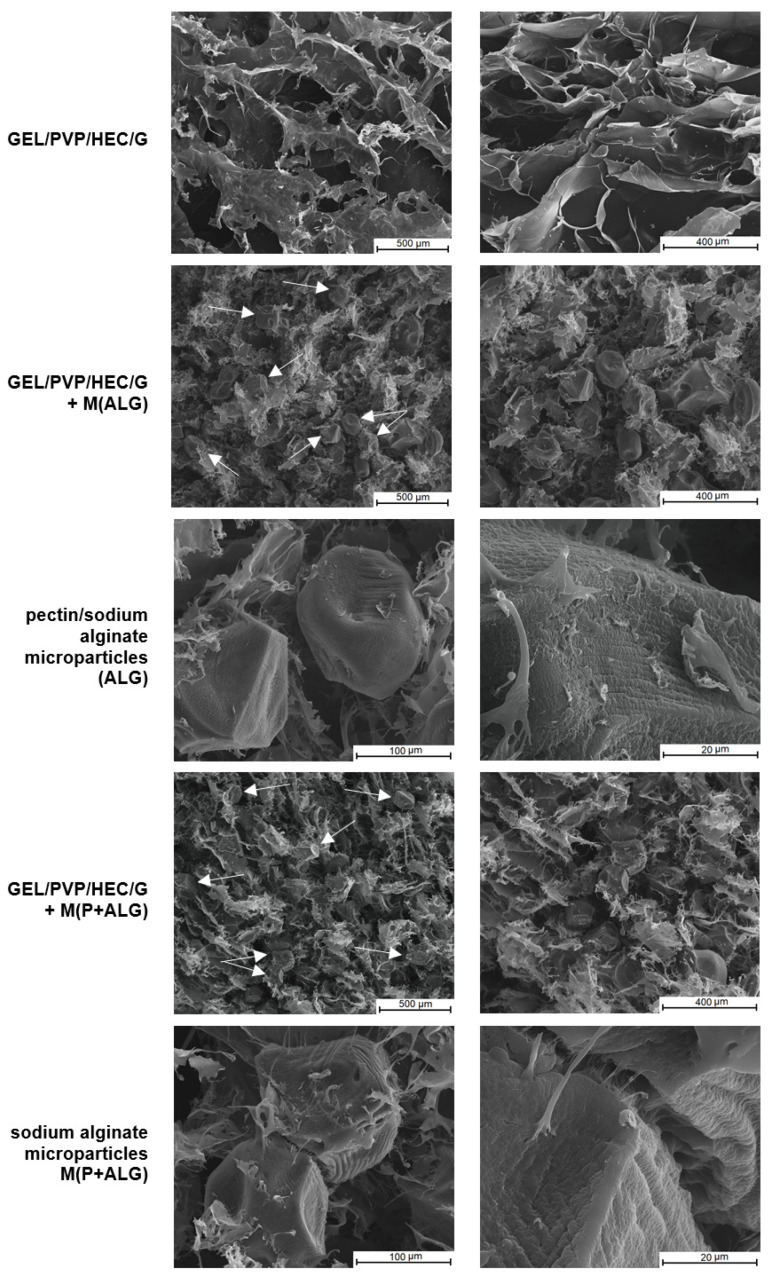
SEM images of the prepared materials with and without microspheres—magnification ×150, ×250 (arrows indicate the location of microspheres in the matrices), SEM images of microparticles in the matrices along with their surfaces—magnification ×1000, ×5000.

The structure and morphology of the obtained sponge-like matrices with and without the addition of microparticles were examined using the scanning electron microscope. The SEM images are presented in Figure 4.

SEM images revealed that the prepared materials had a porous structure with irregular macropores and interconnectivity resulting from the lyophilization of polymer solutions. In this process, the materials are subjected to low temperatures, which leads to the formation of ice crystals and their sublimation under reduced pressure, yielding a porous dry product [56]. Another important observation from SEM images is that sodium alginate and pectin-sodium alginate microparticles were randomly distributed in the matrices, forming clusters in some places. It was noticed that the microspheres filled the pores in the matrices and are weakly connected to the matrix by fiber-like materials. Apart from this, the surface and shape of the freeze-drying microspheres were conscientiously investigated. The obtained microparticles had a ridged and rugged surface with dimples, with a slightly deformed shape, regardless of the microsphere type.

#### 3.2.2. Porosity and Density of Materials

The porosity and density of the prepared materials were assessed by a liquid displacement method, and the results are depicted in Table 2. The freeze-drying technique allows the fabrication of polymer materials with a porous structure and high pore interconnectivity [57].

Based on the presented data, all specimens showed high porosity, about 70% (Table 2). The introduction of the microparticles to the polymer matrix caused the reduction in porosity by about 8% and the increase in the density approximately twice of the studied matrices. This suggested that the microspheres were probably bound to the matrices components and filled pores in the matrices (Table 2). Regardless of the microspheres type, the changes in these parameters were similar. In our previous study, the matrices composed of collagen and hydroxyethyl cellulose with microspheres showed similar properties, i.e., the incorporation of microparticles into polymer matrices led to the decrease in porosity and the increase in sample density [58].

#### 3.2.3. Swelling Tests

**Figure 5:**
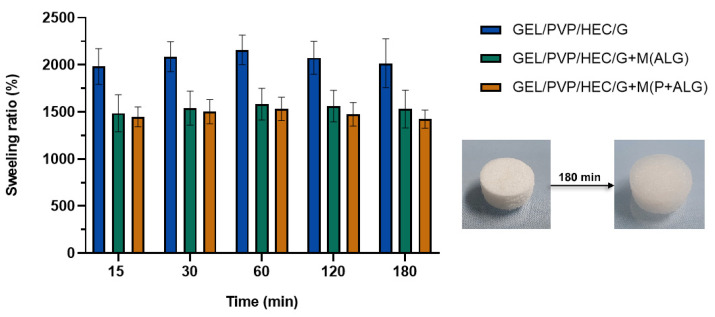
Swelling percentage of GEL/PVP/HEC/G matrices with incorporated microparticles.

Swelling is a physical process in which a material absorbs liquid, increases in volume and mass while maintaining its shape [59]. The swelling test results of the matrices based on gelatin, hydroxyethyl cellulose, polyvinylpyrrolidone, and glycerol with the microparticle addition are shown in Figure 5.

The analysis demonstrated that the obtained materials indicated a high swelling degree (Figure 5). After 60 min of incubation in PBS buffer, they achieved the maximum water uptake capacity. The highest degree of swelling was noticed for the matrix without microparticles (approximately a maximum of 2200%). In contrast, the samples with the addition of microspheres, regardless of their type, swelled less by about one-quarter than the original matrix (with the maximum of 1500% swelling ratio). Thus, the microparticles-loaded materials, due to their higher density and lower porosity, swelled to a lesser extent. Moreover, the water uptake capacity did not differ much for the samples with the microparticles in the studied range of swelling time. Mishra et al. fabricated lyophilized gelatin-PVP scaffold and measured the swelling degree in PBS at different pH (2.5, 7.4, and 9). The polymer composites showed enhanced water uptake in all conditions [60]. Materials with a porous structure, based on hydrophilic polymers such as gelatin and hydroxyethyl cellulose, are characterized by high water absorption ability [61]. Water molecules penetrate the polymer network and hydrate the most polar hydrophilic groups, which are hydroxyl and carboxylic groups, until reached the equilibrium state. The new hydrogen bonds are formed between solvent molecules and the functional groups of the polymeric chain. This stabilizes the structure [59,62].

#### 3.2.4. Dissolution of Matrices

**Figure 6:**
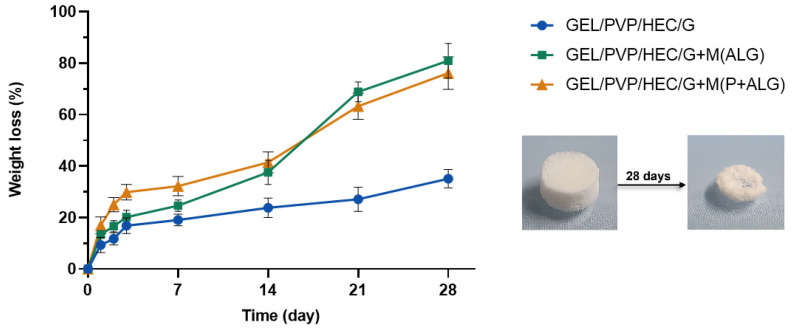
The weight loss during the degradation of the prepared matrices containing the microparticles with the surfactant.

The prepared polymer matrices were placed in PBS solution to investigate the samples’ degradation. Figure 6 shows the weight loss of the specimen versus incubation time in phosphate saline buffer. The degradation ability of polymeric materials depends on their structure, conformation, porosity, and degree of the inter-structure network [63].

The degradation of the matrices containing the microparticles was much faster than the control sample. It could be due to the fact that the microparticles were dissolved or washed out during the 14 days of measurement, which can be seen in the graph as a spike in the weight loss curve. This suggests that the microspheres were weakly associated with the matrix components and entrapped within its structure, and thus degraded faster. The matrix without the microparticles had the highest resistance to dissolution, as after 28 days, the weight loss was about 30%, while the weight loss of the microspheres-loaded matrices was about 80%. This meant that the prepared polymer matrices, with the addition of microparticles with surfactant, dissolved efficiently, which is an advantage for the potential usage of such materials as a degradable substitute for wet wipes.

#### 3.2.5. Mechanical Properties

The results of Young’s modulus, toughness, and yield strength measurements during the compression of polymer matrices under different conditions (dried and soaked in PBS solution) are shown in Table 3.

The prepared materials did not fracture after compression, and they were compacted and flattened. The matrices responded effectively to the applied stress, absorbing energy and dissipating it without fracture.

The introduction of the microparticles to the polymeric matrices resulted in the higher stiffness and toughness of the microparticles-loaded samples. It was evidenced by higher values of Young’s modulus and toughness of the dry and soaked samples compared to the neat matrix (Table 3). As the microparticles embedded in the pores of the polymeric matrix, they stiffened and toughened the matrix structure. This indicates that GEL/PVP/HEC/G matrix was more flexible. Moreover, the type of microparticles had no significant effect on the mechanical properties of the matrices. Nooeaid et al. prepared porous gelatin scaffolds using lyophilization with the addition of hydrochloride-encapsulated polylactic acid microparticles. The published data showed that the elastic modulus value of gelatin matrices was about 500 kPa, and the incorporation of microparticles into these scaffolds increased the value of elastic modulus [64]. The yield strength was determined for the dry materials. The yield point value of the matrices with microparticles was twice or about thrice higher than that for the neat material. This indicates that the ability to plastic deformations was achieved with a lower stress value for the control sample than for microparticle-loaded matrices. As expected, the submergence of the studied samples in the buffer led to a significant decrease in the compressive modulus and toughness due to their efficient hydration. Consequently, the swollen specimens were much less rigid and tough than the dried materials. Water molecules penetrate the matrices making the studied matrices very soft.

#### 3.2.6. Thermal Analysis

**Figure 7:**
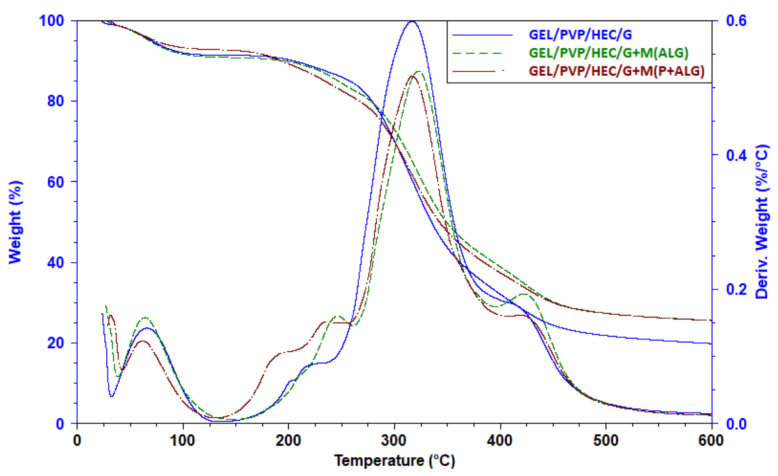
Thermogravimetric (TG) and derivative thermogravimetric (DTG) curves of GEL/PVP/HEC/G matrices with and without microparticles.

Figure 7 shows TG and DTG curves of the polymeric matrix and the matrices with the microparticles. The first stage of decomposition of the sample at a temperature below 110 °C observed for all tested samples was related to the moisture release. All samples lost about 7–9% of their weight.

The main degradation stage started at 150 °C in the case of the neat matrix and the matrix with alginate microparticles, whereas for the matrix with pectin-sodium alginate microparticles, this process started at a lower temperature, probably due to the lower thermal stability of pectin. This degradation stage was complex, and at least three different processes overlapped as shoulders in the 150–200 °C temperature range and a shoulder at about 420 °C were observed. In this stage, the samples lost about 63–70% of their weight (Table 4). The most efficient decomposition process was detected for the control sample, while this process was less efficient for the matrix loaded with microparticles.

Thermal degradation often results in not total sample decomposition as a carbon residue can be found in a crucible after heating. It means that the sample material underwent thermal crosslinking. The carbon residue at 600 °C was higher for the matrices with the microparticles indicating that the polymers, from which the particles were made, took part in the thermal crosslinking. This process was more efficient in the presence of sodium alginate and pectin. Thus, lower weight loss in the main stage for these samples resulted from the thermal crosslinking of the polymers.

## 4. Conclusions

The project aimed to find a degradable replacement for wet wipes. For this purpose, the 3D sponge-like matrices and microparticles filled with coco-glucoside were produced from degradable, water-soluble polymers. The matrices were obtained from gelatin, PVP, and hydroxyethyl cellulose, whereas the microparticles were made of alginate and pectin. Furthermore, the surfactant of natural origin was used.

The research showed a slightly larger size and narrower size distribution of the spherical microparticles composed of sodium alginate and pectin compared with the microparticles obtained from sodium alginate. The prepared materials had high porosity, swelling capacity, and good dissolution in PBS. The SEM images confirmed the microparticles’ location in the matrices. The reduction in porosity and ability to absorb water in the samples with the microparticles due to the filling of pores in the matrices by the microspheres was also noticed. Moreover, a faster degradation rate in the PBS buffer of the composites with the microparticles was observed owing to removing the microspheres from the sample and then better access of the solution to the matrix. Mechanical properties tests exhibited an increase in Young’s modulus and toughness for matrices containing microparticles due to the stiffening of these materials. Thermal analysis showed that the polymers forming microparticles took part in the polymer thermal crosslinking, resulting in less weight loss in the main degradation stage and a higher carbon residue in these samples. The effect of the microspheres on the matrix properties was similar, regardless of the type of microparticles.

## Figures and Tables

**Table 1:** Characteristics of the prepared microparticles. X_10_, X_50_, and X_90_ represent the volume percentages of particles (10%, 50%, and 90% undersize, respectively).

Microparticles	Mean Particle Size (µm ± SD)	Particle Size (µm)			Span
		X_10_	X_50_	X_90_	
sodium alginate M(ALG)	474.9 ± 0.112	342.7	482.2	663.3	0.6647
pectin-sodium alginate M(P + ALG)	560.7 ± 0.094	412.4	559.2	742.8	0.5908

**Table 2:** Porosity (Є) and density (*d*) of GEL/PVP/HEC/G matrices containing microspheres (values with standard deviation).

Sample	Є (%)	*d* (mg/cm^3^)
GEL/PVP/HEC/G	73.3 ± 7.7	23.1 ± 5.6
GEL/PVP/HEC/G + M(ALG)	65.1 ± 4.2	44.0 ± 4.5
GEL/PVP/HEC/G + M(P + ALG)	66.3 ± 7.1	39.1 ± 6.9

**Table 3:** The values of Young’s modulus, toughness, and yield strength of the prepared materials under different conditions—dried and after submerged in PBS solution (values with standard deviation).

Sample	Young’s Modulus (kPa)		Toughness (kJ/m^3^)		Yield Strength (N/mm^2^)
	Dry	Soaked	Dry	Soaked	Dry
GEL/PVP/HEC/G	527 ± 86	7.6 ± 1.4	50.2 ± 4.4	1.8 ± 0.7	0.1031 ± 0.01
GEL/PVP/HEC/G + M(ALG)	716 ± 122	12.8 ± 1.7	71.2 ± 8.1	4.8 ± 1.1	0.2257 ± 0.03
GEL/PVP/HEC/G + M(P + ALG)	743 ± 137	14.1 ± 1.2	79.7 ± 8.6	8.4 ± 1.9	0.2986 ± 0.03

**Table 4:** The temperature of the beginning of the sample decomposition (T_0_), the temperature at the maximal rate of the process (T_max_), the weight loss in the main stage (Δm), and the char residue at 600 °C for the prepared materials.

Sample	T_0_/T_max_ (°C)	Δm (%)	Residue (%) at 600 °C
GEL/PVP/HEC/G	150/315	69.56	19.86
GEL/PVP/HEC/G + M(ALG)	150/323	63.41	25.57
GEL/PVP/HEC/G + M(P + ALG)	133/316	65.37	25.57

## Data Availability

Data sharing not applicable.
